# Asymmetric Bilateral Lichen Striatus: A Rare Presentation following Multiple Blaschko's Lines

**DOI:** 10.1155/2018/6905175

**Published:** 2018-06-10

**Authors:** Jeffrey S. Dickman, McKay D. Frandsen, Andrew J. Racette

**Affiliations:** ^1^Midwestern University, Arizona College of Osteopathic Medicine, 19555 N 59^th^ Ave, Glendale, AZ 85308, USA; ^2^Omni Dermatology, Inc., KCU-GMEC Phoenix Dermatology Residency Program, 4840 E Indian School Rd, Suite 102, Phoenix, AZ 85018, USA

## Abstract

Lichen striatus (LS) is an uncommon, acquired, self-limited, and benign linear dermatosis of unknown etiology that most often occurs unilaterally and is confined to the lines of Blaschko. A healthy 7-year-old girl presented to our clinic with bilateral asymmetric LS occurring on the right arm and left leg of 1-year duration. Very few cases of bilateral LS have been previously reported in the literature, with none from clinics within the United States. The etiology of LS is currently unknown; however its confinement to Blaschko's lines, which represent embryologic migration of skin cell clones, does provide insight into a possible pathogenesis. It seems most likely that an individual's development of LS is linked to their genetic predisposition and a subsequent triggering event. Our case serves as a strong example of a rare presentation of LS and facilitates discussion of the clinical diagnostic process and possible pathogenesis of this dermatosis.

## 1. Introduction

Lichen striatus (LS) is an uncommon linear dermatosis that most commonly affects children aged 4 months to 15 years and is distributed along the lines of Blaschko. Diagnosis is made based on clinical appearance of 2 to 4 mm, flat-topped, lichenoid papules ranging from red color to flesh color that are distributed linearly and may be discrete or confluent [1, 2]. A variant presentation may more commonly present with hypochromic macules that are singular or coalesce into a patch [2]. Classically, LS occurs unilaterally and along a singular Blaschko line (BL) typically on the extremities, but a few rare cases have been found occurring bilaterally. To the best of our knowledge less than ten bilateral presentations have been previously reported in the literature, making our patient very unique [3–7]. The etiology of LS remains unclear, though the lesions are benign and the condition is self-limited. Some have hypothesized that the LS may develop in a genetically predisposed individual who encounters an immunologic trigger [1, 8].

In this article we present a patient with bilateral asymmetric LS who reported gradual onset one year prior to presentation. Very few cases of bilateral LS have been previously reported in the literature; and to the best of our knowledge none was from clinics within the United States of America [3–7].

## 2. Case Presentation

A healthy 7-year-old girl of Indian descent presented with one-year duration of hypochromic linear bands in two regions. The lesions were present on the right forearm and left leg and buttocks. Neither the patient nor her parents were able to recall any inciting illness, allergy, or environmental or social exposure that may have preceded the onset, which was gradual. There was no associated pruritus, pain, hair loss, or nail involvement. No recent growth had been noted. The patient had not received any previous topical or systemic treatment for the lesions. The patient's past medical history was negative for atopy and otherwise unremarkable, as was her family history.

**Figure 1:**
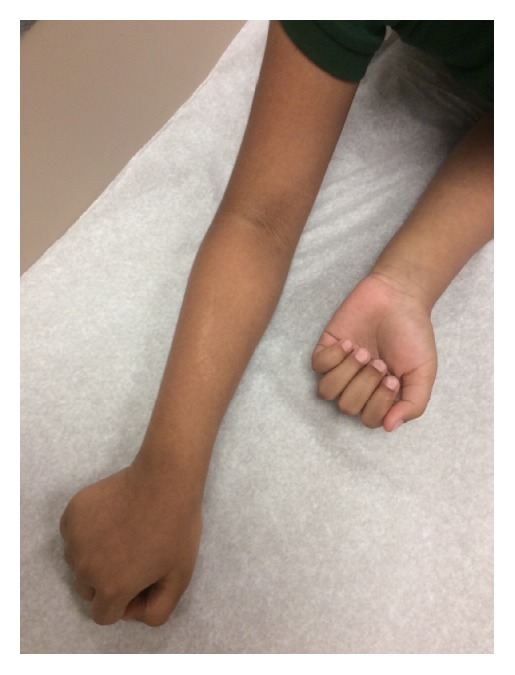
Hypopigmented linear eruption on the right forearm.

**Figure 2:**
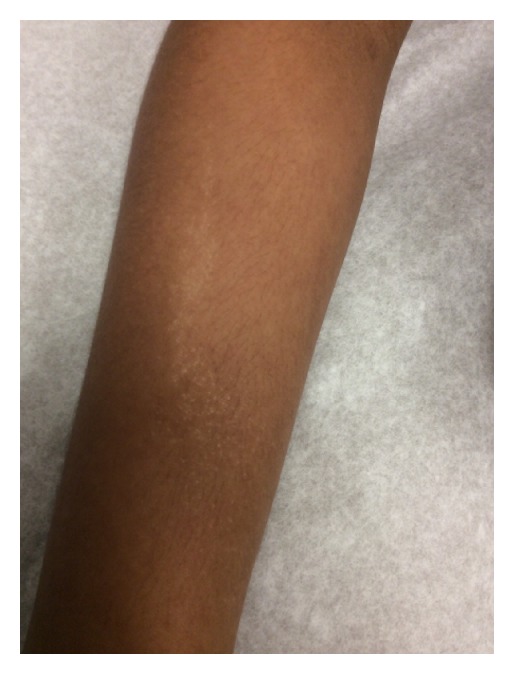
Hypopigmented macules coalescing into patch along BL.

**Figure 3:**
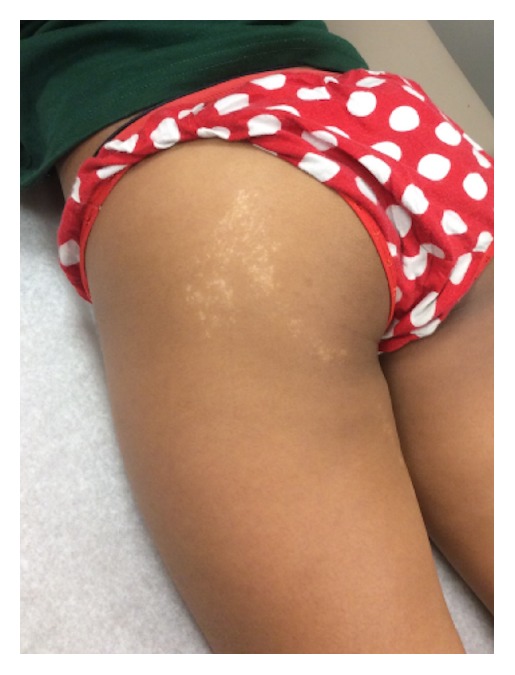
Hypopigmented macules and patch following the lines of Blaschko.

**Figure 4:**
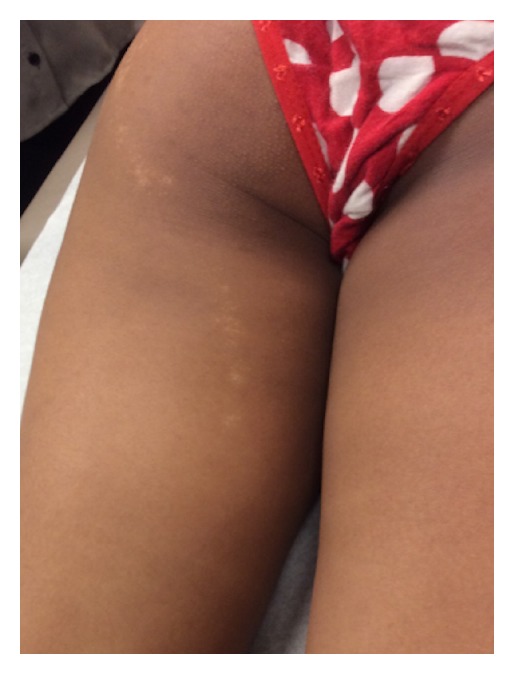
Extension of eruption along the left posterior thigh.

On examination 2 mm hypopigmented lichenoid macules were noted coalescing into a linear patch on the dorsal aspect of the patient's right forearm (Figures 1 and 2). The eruption ended at the distal forearm, sparing the right hand, fingers, and nails. The distribution was consistent with BL. Similar lesions were also noted on the left buttock, though somewhat more diffuse, but also progressing distally along a BL to the left posterior thigh (Figures 3 and 4). The lesions in both locations were nonscaling, nonpainful, nonpruritic, and stable in appearance according to the patient's parents.

No biopsies were taken at the request of the patient's parents. A diagnosis of LS was made clinically, and observation was recommended with explanation of the disease course. A follow-up visit was scheduled but the patient did not return to the clinic.

## 3. Discussion

Lichen striatus is an acquired, self-limited, benign dermatosis of unknown etiology that most commonly occurs unilaterally and is confined to the lines of Blaschko. Its diagnosis can be made based on clinical presentation alone, but careful consideration of other linear eruptions must be given (Table 1) [1, 8–10]. Biopsy and histopathological analysis, when tolerable to the patient, may help to distinguish LS from other lesions but are somewhat nonspecific. Typical histological findings include spongiotic and lichenoid interface dermatitis with superficial and deep perivascular infiltrate and epidermal changes including hyperkeratosis, parakeratosis, focal spongiosis, and lymphocytic exocytosis. The deep lymphohistiocytic infiltrates are also seen surrounding adnexal structures such as hair follicles and eccrine glands [11–13].

Three morphological variants of LS have been described [2]. Typical lichen striatus is most common, presenting as 2 to 4 mm, flat-topped, lichenoid papules ranging in color from red to flesh-colored. This accounts for approximately 80% of patients. Lichen striatus albus presents with hypopigmented macules and/or papules that coalesce into a patch as seen in our patient. The final variant is nail lichen striatus, which in addition to cutaneous lesions affects the nail matrix of usually a single digit. LS in all its forms is more common in females with a ratio of 2:1 [1, 2]. Of note, four of the six comparable cases (including ours) of bilateral LS have occurred in patients of Indian descent [3–5]. A recent analysis performed in an outpatient dermatology department in South India showed that LS made up 1.77% of presenting hypopigmentary disorders [14]. Because the pathogenesis of LS is poorly understood more research is necessary to determine if this association is incidental.

The etiology of LS remains unclear. Its confinement to BL, representing embryologic migration of skin cell clones, does however provide insight into a possible pathogenesis. It has been suggested that a postzygotic somatic mutation followed by an immunologic response directed at these clonal cells may be the cause [11, 15]. Happle later proposed the theory that transposable elements or retrotransposons within the human genome, which affect the activation or silencing of genes, could cause linear skin lesions following BL. This model had been demonstrated in the variegated coat patterns resembling BL in animals [16]. It seems most likely that an individual's development of LS is linked to their genetic predisposition and a subsequent triggering event. It remains unclear however if that event is the activation or suppression of a gene, an immunological response against previously mutated cells, or the result of some external agent.

Our patient is especially interesting because the dermatosis was bilateral. Very few cases with bilateral distribution have been reported in the literature [3–7]. While the disease itself is benign and self-limited, it may present a diagnostic challenge and its pathogenesis is complex. Exploration of additional cases like ours will help us to better diagnose and understand this disease.

## Figures and Tables

**Table 1:** LS differential diagnosis [1, 8–10].

Inflammatory linear verrucous epidermal nevus
Linear epidermal nevus
Linear psoriasis
Linear lichen planus
Linear verruca plana
Linear porokeratosis
Linear Darier's disease
Blaschkitis
